# Antarctic last interglacial isotope peak in response to sea ice retreat not ice-sheet collapse

**DOI:** 10.1038/ncomms12293

**Published:** 2016-08-16

**Authors:** Max D. Holloway, Louise C. Sime, Joy S. Singarayer, Julia C. Tindall, Pete Bunch, Paul J. Valdes

**Affiliations:** 1 Ice Dynamics and Paleoclimate, British Antarctic Survey, Cambridge CB3 0ET, UK; 2 School of Geographical Sciences, University of Bristol, Bristol BS8 1SS, UK; 3 Department of Meteorology, University of Reading, Reading RG6 6BB, UK; 4 School of Earth and Environment, University of Leeds, Leeds LS2 9JT, UK; 5 Department of Engineering, University of Cambridge, Cambridge CB2 1PZ, UK

## Abstract

Several studies have suggested that sea-level rise during the last interglacial implies retreat of the West Antarctic Ice Sheet (WAIS). The prevalent hypothesis is that the retreat coincided with the peak Antarctic temperature and stable water isotope values from 128,000 years ago (128 ka); very early in the last interglacial. Here, by analysing climate model simulations of last interglacial WAIS loss featuring water isotopes, we show instead that the isotopic response to WAIS loss is in opposition to the isotopic evidence at 128 ka. Instead, a reduction in winter sea ice area of 65±7% fully explains the 128 ka ice core evidence. Our finding of a marked retreat of the sea ice at 128 ka demonstrates the sensitivity of Antarctic sea ice extent to climate warming.

## Abstract (web summary)

The peak in Antarctic ice core isotope values, 128,000 years before present, was concurrent with a significantly warmer-than-present Antarctic climate. Here, the authors show that this isotope maximum was associated with a major retreat of sea ice and not a collapse of the West Antarctic Ice Sheet.

## Graphical abstract

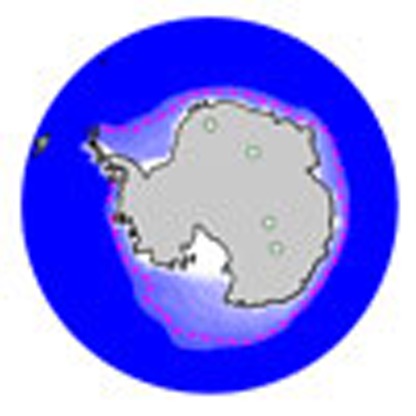

During the last interglacial (LIG; 130,000–115,000 years ago) global climate was warmer than today^1^,^2^,^3^,^4^ and global mean sea level was 6-9 m higher^5^,^6^,^7^,^8^,^9^,^10^ (Fig. 1). This LIG sea-level high stand was mainly driven by ice-sheet loss^5^,^11^. Recent ice core results indicate that the Greenland ice sheet likely provided a modest 2 m contribution towards the global sea-level rise^5^, with estimates ranging from +1.4 m to +4.3 m^12^. This implies that ice loss from the West Antarctic Ice Sheet (WAIS) must have contributed to the LIG sea-level maxima: loss of the entire WAIS would contribute 3–4 m of global sea-level rise^13^,^14^. Coral records from Western Australia indicate that the sea level rose late in the interglacial, ∼118,000 years ago (118 kyr ago)^9^. However, Seychelles coral has been interpreted as indication of a +5 m global sea level at 128 ka^6^. These differing interpretations prevent constraint on the timing of WAIS loss, thus reducing the potential to use the LIG to inform the debate on the likelihood of future WAIS loss^11^,^13^,^14^. We therefore turn to the ice core records to push forward the WAIS loss debate.

The recent ice core drilled at WAIS Divide^15^ does not extend back through the LIG; ice that may have been present during the LIG has since been lost through basal melt. However, ice cores extending back throughout the LIG, at a resolution of <200 years per metre of ice^16^, are available from four locations on the East Antarctic Ice Sheet (EAIS; Fig. 1). From west to east these are: EPICA Dronning Maud Land (EDML); Dome F (DF); Vostok; and EPICA Dome C (EDC). These four ice cores all record an isotopic maximum at ∼128 ka, associated with peak Antarctic warmth^1^,^17^,^18^. Relative to the last 3 kyr ago, this LIG isotopic maximum is between 2 and 4‰ in *δ*^18^O. It has been suggested that WAIS loss is required to explain the magnitude of this isotope maximum^4^,^19^. However, an alternative hypothesis exists; that reduced Southern Hemisphere sea ice extent provides an alternative explanation for the 128 ka isotopic maximum^15^,^20^,^21^,^22^. Both ice and ocean core evidence suggests that a large retreat of the Antarctic sea ice edge may have occurred at 128 ka^21^,^22^,^23^.

We carry out a series of climate model experiments incorporating *δ*^18^O (ref. 24). We evaluate experiments including WAIS loss and Southern Hemisphere sea ice retreat at 128 ka against existing Antarctic ice core data (see Methods for full details and Supplementary Table 1 for a full list of experiments). Our results suggest that a full WAIS collapse cannot explain the magnitude or the spatial pattern of the 128 ka *δ*^18^O maximum. Removing the WAIS causes changes in atmospheric circulation and precipitation seasonality that tends to reduce *δ*^18^O. Including WAIS, meltwater reduces *δ*^18^O by freshening the surface ocean, resulting in cooling and sea ice expansion, which does not improve the model–data agreement. A major sea ice retreat of 65±7% increases *δ*^18^O and does result in a good model–data agreement. This finding will have consequences for sea ice projection in a future warmer climate.

## Results

### 128 ka simulations with changes in WAIS morphology

The isotopic response to 128 ka orbital and greenhouse gas forcing alone (and no change in WAIS morphology; Supplementary Figure 1) is weak (Fig. 2a). Simulated *δ*^18^O anomalies at the ice core sites range between −1.55 and +0.26‰ relative to a pre-industrial (PI) control experiment. When the response to a remnant 200 m WAIS is simulated, *δ*^18^O anomalies at the ice core sites range from −0.18 to +0.96‰ (Fig. 2b); and when the WAIS is fully removed and new ocean regions created, the simulated *δ*^18^O anomalies become further depleted to between −2.78 and +0.63‰ (Fig. 2c). Simulated *δ*^18^O anomalies are strongly positive over the WAIS for all experiments with a reduced WAIS. Reduced elevation increases surface air temperature at a rate roughly proportional to the lapse rate (∼6 °C km^−1^; Supplementary Fig. 2), which in turn enriches the isotopic composition of local vapour. If we include the effects of meltwater from a WAIS collapse, the *δ*^18^O depletion becomes more pronounced (Fig. 2d). A reduction in the Southern Ocean source water *δ*^18^O alongside an expansion in sea ice both tend to reduce *δ*^18^O at the ice core sites. These simulated *δ*^18^O results, from each of our three WAIS loss scenarios, do not match the 128 ka *δ*^18^O values from the ice core data.

### Decomposition of changes in *δ* ^18^O

At the ice core sites, changes in both the isotopic composition and the seasonality of precipitation contribute to the simulated negative *δ*^18^O anomalies. Although the precipitation over the ice core sites tends to be enriched during colder months due to WAIS loss, an increased proportion of precipitation falling during colder months leads to an overall depletion of *δ*^18^O (Supplementary Figs 3 and 4).

To qualify the relative impact of precipitation and *δ*^18^O seasonality, we first isolate the changes in *δ*^18^O due to the changes in the seasonal cycle of precipitation (Δ*P*_seas_)^25^;


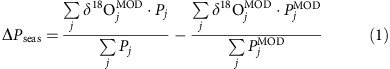


Superscript ^MOD^ indicate values from the 128 ka experiment using a modern WAIS configuration and no superscript indicate values from the WAIS sensitivity experiments. The difference between the total *δ*^18^O change (Δ*δ*^18^O) and Δ*P*_seas_ represents other effects contributing to the observed *δ*^18^O anomaly (such as variability in the *δ*^18^O of precipitate and in the source vapour);


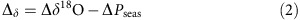


For all WAIS retreat scenarios (a remnant WAIS, WAIS removed and replaced with ocean, and WAIS removed and meltwater added to the Southern Ocean) Δ*P*_seas_ is negative; a larger proportion of precipitation falls on the EAIS during cold months when the WAIS is absent (Fig. 3b,e,h). This differs from the WAIS loss experiments of Holden *et al.*^19^, who observe an increase in summer precipitation. This discrepancy is likely explained by differences in the modelling set-up; Holden *et al.* include different boundary forcing (chosen for 130 kyr ago), the WAIS replaced by ‘ice-free' land at an elevation of 200 m, and 1 Sv of freshwater added to the North Atlantic.

Changes in Δ_*δ*_ are strongly positive over the WAIS for all experiments with a reduced WAIS, which is a direct response to the lowered elevation and associated warming, mentioned above (Fig. 3c,f,i). The response of Δ_*δ*_ over the EAIS differs between the WAIS retreat scenarios. Δ_*δ*_ is positive over the EAIS for a remnant flat WAIS, but turns negative when the WAIS is removed and replaced with ocean. This suggests that there are changes in the intensity of precipitation falling over the EAIS and/or a change in precipitation source region when the WAIS is replaced with ocean. Such changes in the amount and/or intensity of precipitation over Antarctica would be consistent with the expected changes in the thermal characteristics of the high southern latitudes; lower AIS topography has been linked with intensified cyclones over the continent (suppressed for higher AIS)^26^. These changes allow more storms to travel over the continent, which are a key mechanism for transporting moisture inland^26^.

When the WAIS is replaced with ocean and meltwater is added to the Southern Ocean, Δ_*δ*_ is negative everywhere apart from the elevation-induced-positive anomalies over the WAIS (Fig. 3i). This is a response to the depleted isotopic composition of the prescribed meltwater (-30‰), depleting the isotopic composition of the surface Southern Ocean that is a source for Antarctic precipitation, and a freshwater-associated expansion in Southern Hemisphere sea ice.

### 128 ka simulations with WAIS and sea ice retreat

Sea ice retreat in the presence and absence of the WAIS both enrich *δ*^18^O at the ice core sites. Water vapour becomes relatively enriched in heavy isotopes in response to the evaporative input from new water surfaces exposed by the retreat of sea ice. A reduced distance between evaporation source and precipitation site for atmospheric water vapour tends to enrich *δ*^18^O^20^. However, there are considerable differences across East Antarctica in the *δ*^18^O response to WAIS presence and WAIS loss. Following a Bayesian analysis, we assess which of these scenarios best explains the observed data (see Methods for details). Our results strongly support the conclusion that the WAIS was present at 128 ka. Comparing the two scenarios using a statistical model comparison, the likelihood ratio is 200 in favour of the WAIS being present, that is, the observations are 200 times more likely using a model with the WAIS present than when the WAIS is removed. The WAIS removed scenario does not explain the observed spatial pattern of *δ*^18^O measurements, as well as the model simulations that retain the WAIS.

When the WAIS is present, a winter (September) sea ice area reduction of 65% (posterior mean with a 95% credibility interval of 58–72%) relative to pre-industrial provides a data–model match of better than ±0.02‰ with the *δ*^18^O anomaly at Vostok and EDML, better than ±0.8‰ at EDC and ±1.1‰ at DF (Figs 4a and 5). With the WAIS removed, the best fit to the ice core observations is similarly achieved with a sea ice reduction of 66%. However, the uncertainty band is nearly four times larger (95% credible interval of 32–87%) and the model–data match is worse at every site; the model–data *δ*^18^O match is worse than ±0.05‰ at EDML, ±1.0‰ at Vostok, ±1.9‰ at EDC and ±3.5‰ at DF (Fig. 4b). This multi-ice core data–model comparison thus suggests that complete loss of the WAIS at 128 ka is inconsistent with the ice core evidence.

## Discussion

**Figure 1:**
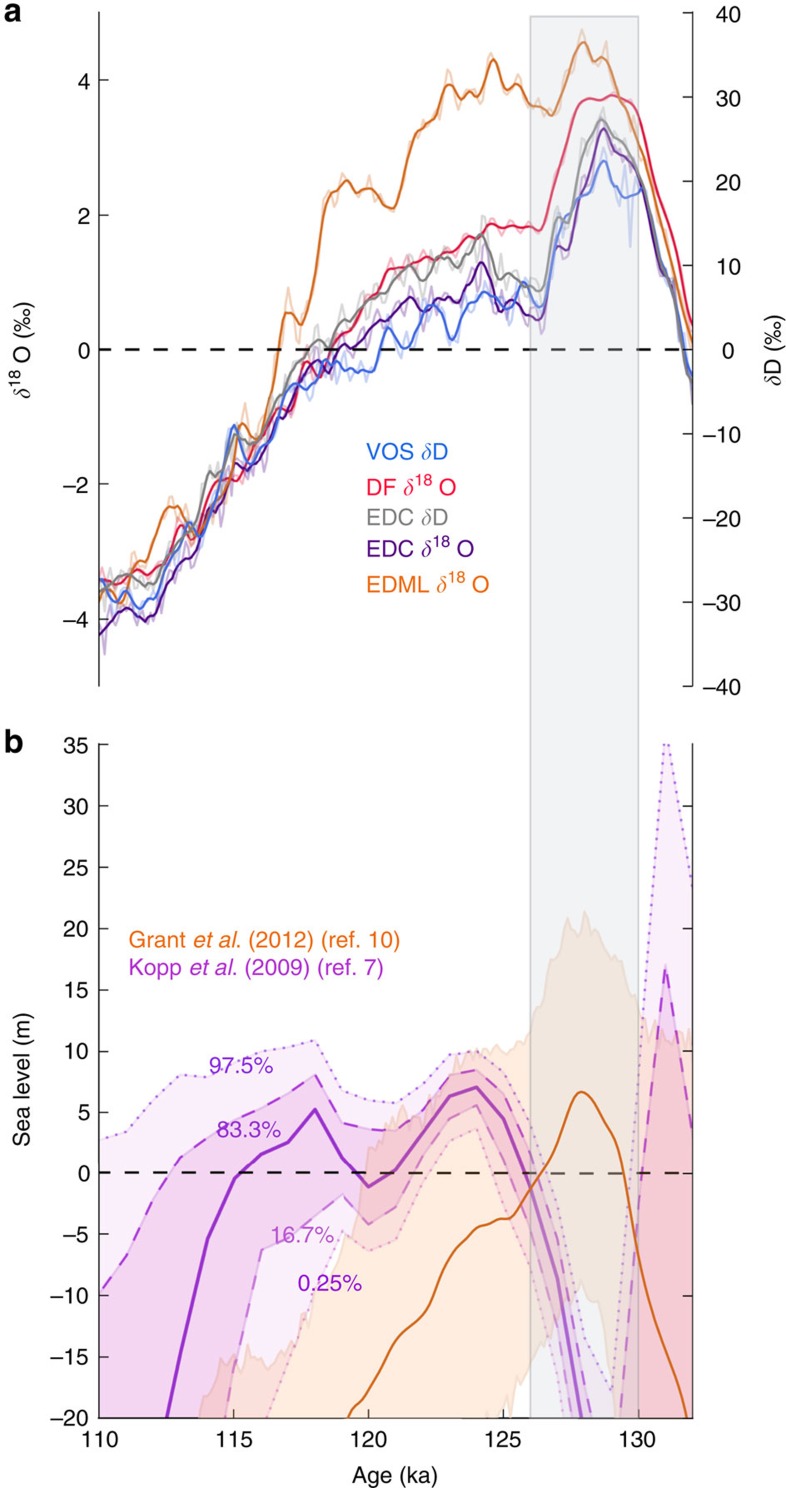
Time series of Antarctic ice core stable water isotope records and sea level during the last interglacial. (**a**) Stable water isotope (*δ*^18^O and *δ*D) anomalies relative to the last 3 kyr ago from four deep ice cores^16^: Vostok (blue), Dome F (DF; red), EPICA Dome C (EDC; grey for *δ*D and purple for *δ*^18^O) and EPICA Dronning Maud Land (EDML; orange). (**b**) Global sea level^7^ (purple curve; heavy line marks median projection, dashed lines the 16th and 84th percentiles, and dotted lines the 2.5th and 97.5th percentiles) and Red Sea relative sea-level^10^ records (brown curve; solid line shows maximum likelihood and shading represents 95% confidence limits). The Antarctic isotope peak at 128±2 kyr ago is shaded grey.

We have explored only complete WAIS loss, rather than WAIS reduction, scenarios here. Our results thus do not preclude some loss of the WAIS by 128 ka, or that the WAIS may have been lost later in the LIG, possibly preconditioned by the early retreat of Southern Hemisphere sea ice. Indeed, loss of the WAIS between 128 and 125 kyr ago and a meltwater driven build-up of Southern Hemisphere sea ice may provide an explanation for the late LIG *δ*^18^O drop observed in ice core records; the *δ*^18^O trend throughout the early LIG, with a significant peak and subsequent drop, is distinct from the isotope record of the present interglacial (Fig. 1). Our results indicate that the LIG isotope trend may be consistent with a WAIS collapse and sea ice build-up in the following few thousand years of the isotope maximum.

The difference between an isotope record from Mt. Moulton and East Antarctic ice core records^27^ may also be consistent with a slow loss of the WAIS, which could have been mostly melted after another 2,000 years, by ∼126 kyr ago. Lower isotope anomalies in the Mt. Moulton record relative to isotope records from East Antarctica suggest a local cooling anomaly, which is consistent with climate model simulations of WAIS collapse driven by pre-industrial boundary conditions^27^. The low isotope values in the Mt. Moulton record, relative to the other ice core sites, persists throughout the LIG, but the difference is greatest after ∼126 kyr ago, perhaps coinciding with maximum retreat of the WAIS. Considering the reasonable agreement between the observed peak-to-trough *δ*^18^O anomalies and those calculated between our sea ice retreat and the WAIS loss experiments (Supplementary Fig. 5), we suggest that a large sea ice retreat best explains the early isotope maximum and a subsequent retreat of the WAIS, and sea ice build-up could provide an explanation for the observed pattern of isotope anomalies following the LIG maximum.

The bipolar seesaw mechanism^28^ proposes that a slowdown in northwards ocean heat transport, particularly in the Atlantic, tends to warm the Southern Ocean. This mechanism is consistent with a recent bipolar re-interpretation of the early LIG^29^, alongside a recent synthesis of sea surface temperature reconstructions between 40 and 60° S (ref. 3). These all support Southern Ocean warming at 128 ka, providing a partial explanation for why Southern Hemisphere sea ice retreated at 128 ka. In future work, we will investigate whether the bipolar seesaw can provide the mechanism to cause a major Southern Hemisphere sea ice retreat and thus reconcile the 128 ka *δ*^18^O maximum. Further simulations, including WAIS loss and North Atlantic meltwater input, could provide insight into the non-linear interactions between the bipolar seesaw, the WAIS and Southern Hemisphere sea ice.

Finally, we note the similarity between the wintertime sea ice reduction of up to 58% forecast for the end of the 21st century^12^ and our 58–72% decrease suggested for 128 ka. This implies that the 128 ka sea ice retreat may prove a crucial model–data target for the sea ice modelling community. Currently, the most recent Coupled Model Intercomparison Project Phase 5 multi-model simulations^2^,^12^,^30^ do not simulate a reduction in September sea ice area >13% between the LIG and the present interglacial (Supplementary Discussion; Supplementary Table 2). Considering the disagreement between modelled and observed Antarctic sea ice during the satellite era^31^, a number of studies have called for improvements in the modelling of climate and climate change in the Antarctic region^31^,^32^,^33^. Whether this recent discrepancy is a function of natural variability^34^ or represents a failing of current climate models is still a matter of debate^31^. If the currently observed increase in Antarctic sea ice is robust, a major reduction at 128 ka could indicate a tipping point in the sea ice system. There is clearly a need for more (and more robust) data for Antarctica and the surrounding sea ice edge during the LIG. If it is possible to correctly simulate the 128 ka sea ice reduction, it would improve the low confidence associated with future predictions of Southern Hemisphere sea ice change and, subsequently, improve projections of Antarctic temperature, precipitation and mass balance^35^.

## Methods

### Ice core data

Four published ice core records from East Antarctica cover the LIG at a resolution of <200 years per metre of ice^16^: Vostok^36^, DF^37^, EDC^1^ and EDML^39^. Fractional isotopic content is expressed for oxygen-18 as: *δ*^18^O=1,000 × [(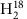

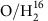
O)/R_VSMOW_−1] (in ‰), where R_VSMOW_ is the ratio of 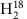
O to 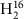
O for Vienna standard mean ocean water. The ice core isotope records are synchronised to the EDC3 age scale^39^ and interpolated onto a common 100 year time grid using an interpolate point method. To minimize the effect of residual temporal misalignment between the ice cores, a 1,500 year low-pass filter is applied to each record before taking the LIG peak^18^. The misalignment and isotope measurement error is then assumed to be negligible after this averaging. The EDC3 age scale was chosen because the version of the EDML record corrected for upstream altitude changes and for the changing *δ*^18^O of seawater is not available on the more recent Antarctic ice core chronology 2012 (AICC2012) age scale. However, as we are only interested in the LIG *δ*^18^O maximum across ice core records, the choice of chronology does not have a significant influence on our results.

### Isotope-enabled general circulation model experiments

The isotope-enabled coupled general circulation model used in this study is the UK Met Office HadCM3 model. HadCM3 has been tested for the present day^24^, the Last Glacial Maximum^40^, as well as warm interglacials of the past^40^,^41^. HadCM3 can be run for multi-millennial length simulations. The model has a reasonable representation of the global distribution of isotopes in the ocean and atmosphere^24^,^41^. Among the Climate Model Intercomparison Project Phase 3 model group, HadCM3 was assigned one of the highest skill scores based on global mean sea-level pressure, sea surface temperature, height and temperature at 500 hPa, and surface mass balance over Antarctica^42^. The effect of seasonal biasing simulated by the HadCM3 model over Antarctica for the present day is similar to that calculated using the ECMWF ERA40 reanalysis product^43^.

We use HadCM3 to simulate the isotopic response to differing WAIS deglaciation scenarios and sea ice retreats during the LIG isotope maximum, 128 ka. We perform three suites of experiments, all forced with orbit and greenhouse gas values for 128 ka and compare to a pre-industrial control simulation, forced by 1850-years before present (BP) orbit and greenhouse gas concentrations. The first suite uses a modern WAIS volume and shape, so the only difference from the control experiment are the 128 ka orbit and greenhouse gas forcing.

A second suite explores the isotopic response to WAIS deglaciation and includes experiments with: (i) a remnant WAIS with elevations reduced to 200 m and ice covered, following the approach of Holden *et al.*^19^; (ii) the WAIS removed and replaced with a new region of ocean of 200 m depth; and (iii) as (ii) but with isotopically depleted meltwater from the WAIS added to the surface Southern Ocean. A prescribed freshwater flux of 0.4 Sv is added over a 100 year simulation (continued from the spun-up WAIS removed simulation), equivalent to a collapse of the WAIS and a global sea-level contribution of 3.5 m. This can be considered an aggressive scenario and represents an idealized catastrophic collapse of the WAIS, such would be required by a very early complete loss of the WAIS during the LIG. The meltwater is distributed over the Southern Ocean according to current iceberg trajectories^44^,^45^. The meltwater is added with an isotopic composition of −30‰, which is approximately equal to that of the parent ice sheet^16^. Apart from (iii), all experiments have been run for at least 700 years. This ensures that the upper ocean and atmosphere are in quasi-equilibrium with the respective boundary conditions. The new regions of ocean that are created when the WAIS is removed are allowed to evolve in the coupled simulation. To our knowledge, these are the first isotope-enabled, coupled atmosphere–ocean global climate model simulations, in which the WAIS has been removed and inundated with ocean. No changes have been applied to the topography of the EAIS. This ensures we isolate the climate response to WAIS changes.

**Figure 2:**
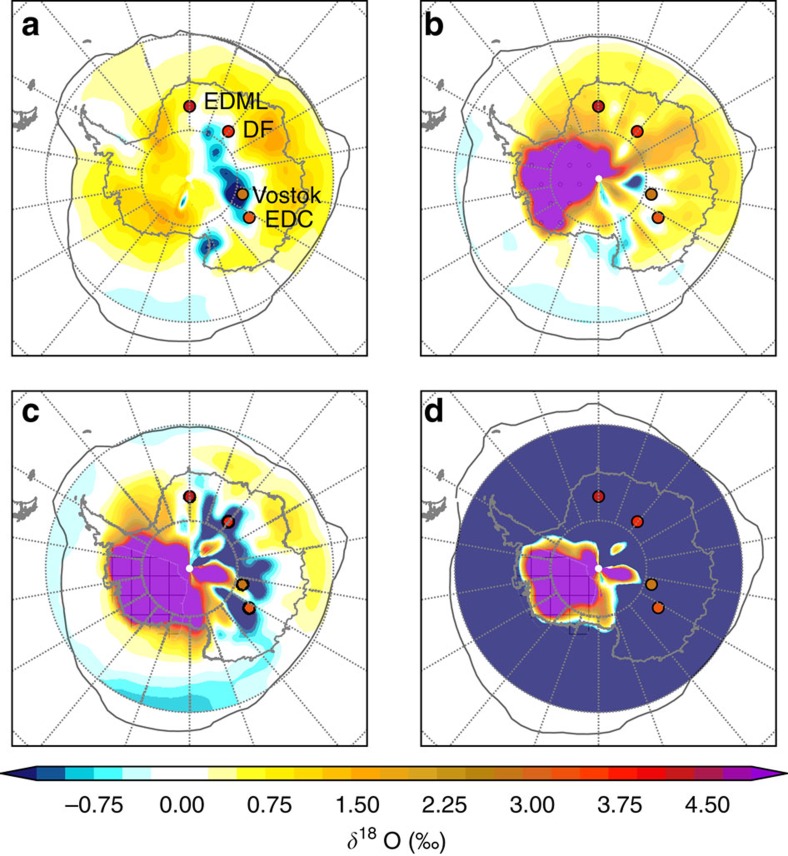
Spatial pattern of *δ*^18^O anomalies. Precipitation weighted *δ*^18^O anomalies relative to a pre-industrial (PI) control experiment (LIG-PI) for 128 kyr ago simulations with (**a**) a modern WAIS configuration, (**b**) the WAIS flattened (indicated by stippling), (**c**) the WAIS removed and replaced with a new region of ocean (indicated by crosshatching), and (**d**) the WAIS removed and meltwater added to the Southern Ocean. Filled circles show ice core *δ*^18^O anomalies for the LIG maximum at ∼128 kyr ago (Methods). Grey lines signify the 15% September sea ice concentration threshold.

**Figure 3:**
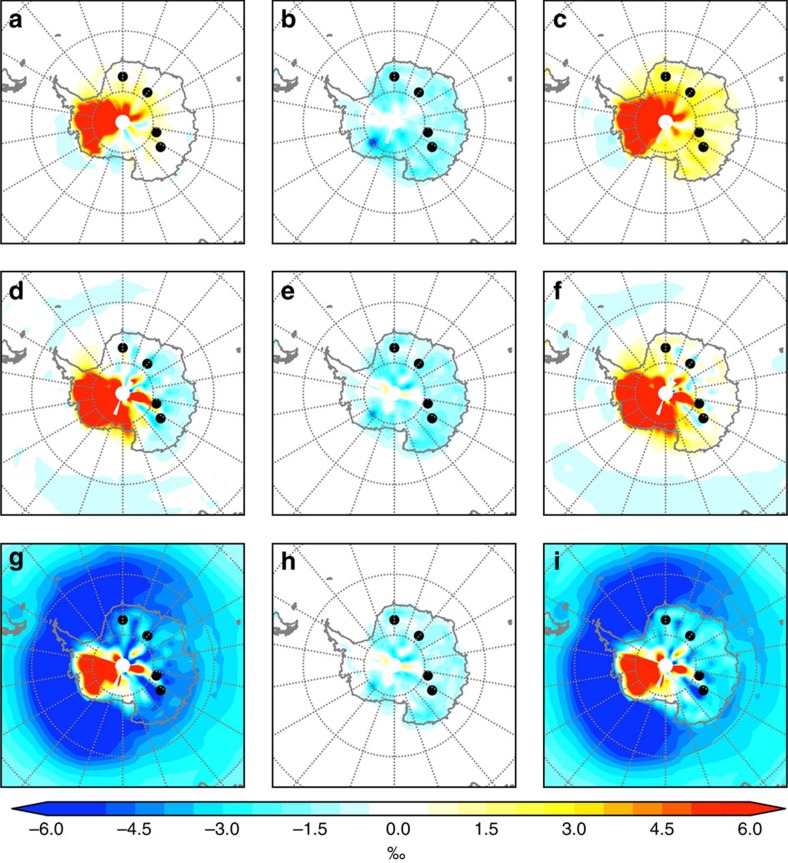
Decomposition of *δ*^18^O anomalies from 128 kyr ago WAIS retreat experiments. (**a**–**c**) A remnant flat WAIS; (**d–f**) WAIS removed and replaced with ocean; (**g–i**) WAIS removed and meltwater added to the surface Southern Ocean. (**a,d,g**) The total *δ*^18^O change between experiments (Δ*δ*^18^O). (**b,e,h**) The change due to the changes in the seasonal cycle of precipitation (Δ*P*_seas_). (**c,f,i**) The change due to other effects, such as the monthly isotopic composition of precipitation (Δ_*δ*_). Anomalies are calculated relative to a 128 kyr ago experiment using a modern WAIS configuration. This calculation was performed using isotopic output from the native model grid, with no re-gridding, due to the need for monthly resolved data (see Methods).

**Figure 4:**
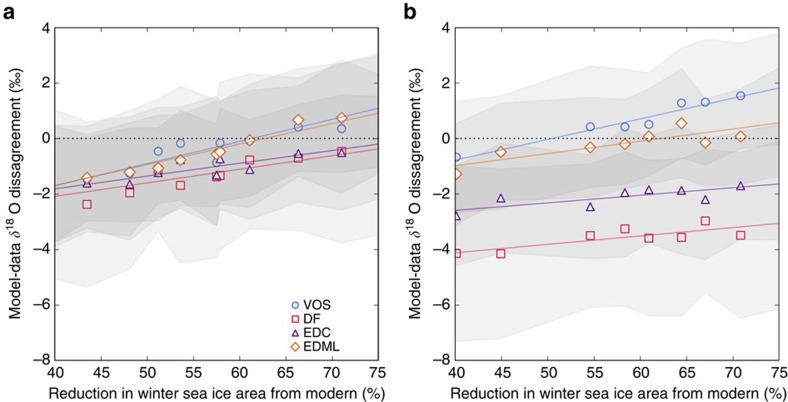
Model–data *δ*^18^O match at ice core sites. Ice core sites shown include Vostok (VOS; blue circles), Dome F (DF; red squares), EDC (purple triangles) and EDML (orange diamonds). Results shown for sea ice retreat experiments and (**a**) a modern WAIS configuration, and (**b**) with the WAIS removed and replaced with ocean. Sea ice retreat is measured as the percentage change in winter (September) sea ice area, relative to the pre-industrial control experiment. Shaded envelopes signify 1 s.d. on simulated annual *δ*^18^O at each site. Best fit lines have been added to each site (coloured as above).

**Figure 5:**
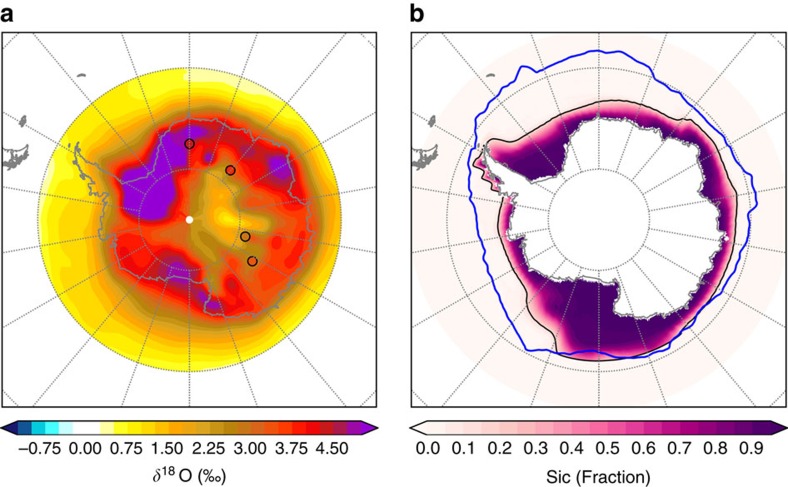
Spatial pattern of *δ*^18^O anomalies for the best fit sea ice retreat. Precipitation weighted *δ*^18^O anomalies (LIG-PI) interpolated between 128 kyr ago experiments to best fit the ice core LIG maximum, corresponding to a 65% winter sea ice retreat relative to pre-industrial. (**b**) September sea ice concentration (sic) fraction, corresponding to **a**. Black contour signifies the simulated 15% September sea ice concentration threshold. Blue contour signifies 1978–2013 satellite observations.

To investigate whether Southern Ocean sea ice retreat can provide an alternative explanation for the LIG isotope maximum, a third suite of experiments are performed using both the modern WAIS configuration, and with the WAIS removed and with a forced reduction in Antarctic sea ice extent. Each experiment is continued from the spun-up 128 ka modern WAIS, and WAIS removed simulations and continued for an additional 50 years. We adopt a ‘clean' method to force a sea ice retreat by prescribing a heat flux to the bottom of Antarctic sea ice at all longitudes and all latitudes south of 49° S, with no other effect to the model physics. The sea ice forcing is held constant throughout the annual cycle, so the model can still calculate the seasonal cycle of sea ice growth and decay. Therefore, the simulated sea ice evolution is only reduced from the coupled models equilibrium response, but still consistent with the internal model physics, and sea surface temperatures and sea ice in our simulations are always internally consistent. The sea ice thus evolves with the coupled model, and the ocean and atmosphere respond to sea ice changes. We perform a range of experiments, each with a different prescribed heat flux from 0 (no forcing) to 120 W m^−2^ (see Supplementary Table 1 for a full list of experiments). The sea ice retreat experiments with the WAIS removed do not include WAIS meltwater added to the surface Southern Ocean, as this scenario was regarded as unrealistic; although it is plausible that sea ice retreat could occur coincident with WAIS retreat and the associated meltwater input, the associated surface freshening and cooling would promote an expansion of Southern Hemisphere sea ice. This is supported by Fig. 2d and Supplementary Table 1, which suggest that the inclusion of meltwater from a WAIS collapse results in a 15% increase in sea ice area. It is therefore more plausible that the meltwater input was not coincident with sea ice retreat.

All modelled isotopic output is first re-gridded to an equal area 50 km grid and smoothed with the surrounding 100 km to remove grid dependence^43^ before evaluation against ice core data. We calculate the simulated s.d.'s, from annually resolved *δ*^18^O model output, and those observed in the ‘raw' ice core records (before being synchronised, placed on a common time scale and filtered; see the previous section). Modelled and observed s.d.'s for each of the four ice core sites (Vostok, DF, EDC and EDML) are 2.18‰, 2.70‰, 1.85‰ and 1.87‰, and 3.31‰, 2.12‰, 2.97‰ and 5.76‰, respectively. We also note the reasonable agreement with results from a high-resolution EDC ice core record, describing the LIG on a 20 year resolution; suggesting a 3,000 year running mean s.d. of 4.5‰^46^.

### Statistics

Inference about the sea ice retreat is conducted using the framework of Bayesian multivariate linear regression^47^. A linear model is first fitted to the simulation outputs. *x*^(*j*)^ denotes the input heat flux for the (*j*)th simulation, 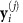 the vector of simulated annual average isotope values at the four measurement sites in the (*i*)th equilibrium year of the (*j*)th simulation, and 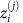 the corresponding sea ice retreat. Here we use the term ‘equilibrium years' to describe the model years after the surface ocean and atmosphere have reached a quasi-equilibrium with the input heat flux, and the sea ice response has converged to a new steady state. The number of simulations is *N*, each of which has *K* equilibrium years.

The sea ice response reaches an equilibrium with the input heat flux within 20 years of each simulation, so we use *K*=30, that is, we use the last 30 years from each 50 year sea ice forcing experiment for the following calculations. We include the experiments with heat fluxes of 30, 35, 40, 45, 50, 60 and 80 W m-2 such that *N*=7. The isotope and sea ice retreat values are modelled as jointly normally distributed with a linear dependence on the input heat flux,


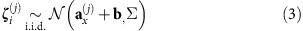


where 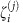 is a vector of all the dependent variables,


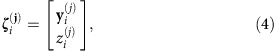


and the unknown model parameters are the slope (**a**), intercept (**b**) and covariance matrix (∑). Note that **a** and **b** are five-element column vectors with the first four elements corresponding to the isotope measurements at the four sites and the fifth corresponding to the sea ice retreat. ∑ is a 5 × 5 positive definite matrix. This can be written equivalently in matrix form using,


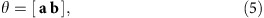



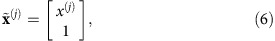


such that


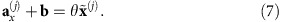


The complete sets of simulation variables will be written as


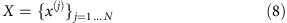



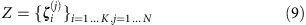


The model makes some strong assumptions about the temporal behaviour of the dependent variables. Over long time periods, climate variables are clearly not well modelled by a constant plus white noise, but display trends and seasonalities. However, over short intervals this simple equilibrium model can be sufficiently accurate. We checked for whiteness by testing all time series (those from the simulations and the equilibrium portions of the isotope records) with a Ljung–Box test^48^, using six lags following the guideline of K/5 (ref. 49), combining *P* values using Fisher's method^50^. There is no significant autocorrelation in the isotope measurements, but the simulation data for sea ice retreat does contain significant values for short lags. To remove this, we apply a preliminary whitening step. For this, we model the raw data as the output of an autoregressive process of order 1 with unknown mean,


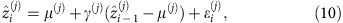


where *μ*^(*j*)^ is the constant mean, and 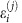 is an i.i.d. Gaussian perturbation. We can transform such a time series to an independent and identically distributed (i.i.d), one using the following transformation,


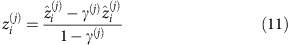



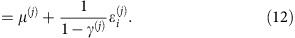


To do this, we first need to estimate *γ*^(*j*)^, which can be achieved using a simple maximum likelihood procedure (jointly with *μ*^(*j*)^). This method allows us to remove temporal correlation, replacing it with an increased variance of each data point conditional on the preceding one. Full details can be found in the supporting iPython notebook.

We can write a probability density for the simulation variables conditional on the parameters,


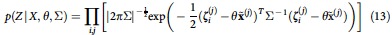



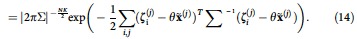


To infer the values of the model parameters, we first assign them a conjugate prior, which is known to be a matrix normal-inverse Wishart distribution^51^,


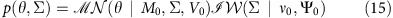



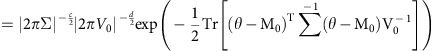



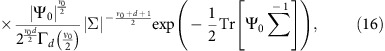


where *c* × *d* are the dimensions of θ, that is, *c*=2, *d*=5 and *M*_0_, *V*_0_, *v*_0_ and Ψ_0_ are hyperparameters to be specified. Since, we have no particular prior information about the parameter values, we choose to make the prior uninformative. We obtain the Jeffreys prior by setting Ψ_0_→0_5 × 5_ (denoting the 5 × 5 matrix of zeros), 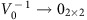 and *v*_0_→0 52. (We could use a weakly informative prior to encode some basic deductions such as the fact that we expect **a**_Z_ to be positive. However, since we have an informative likelihood function for this stage of the inference, the effect of such a prior is practically negligible).

We can combine prior and likelihood to obtain a posterior distribution using Bayes' theorem,


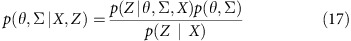



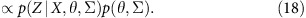


Note that we can ignore the denominator, since it does not depend on *θ* or ∑. The unknown scale factor can be resolved by enforcing that the resulting probability distribution must integrate to 1. Because we chose to use a conjugate prior, the posterior is also a matrix normal-inverse Wishart distribution^51^,


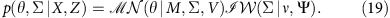


The updated hyperparameters are,


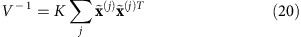



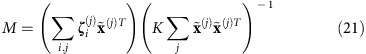



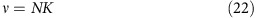



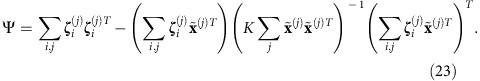


Note that the prior hyperparameters do not appear in these expressions because of our choice of the Jeffreys prior.

The model trained on the simulated data describes the distribution of annual isotope and sea ice retreat values. However, the ice core data does not provide annually resolved measurements. Furthermore the temporal resolution of the various ice cores is not the same, and there is likely to be some residual misalignment in the records even after the records have been synchronised. As stated above, we mitigate these effects by averaging the ice core isotope measurements over a selected interval of *L* years, where *L*=1,500. The chosen value of *L* represents an interval that is as large as possible, while not compromising the assumption that the system is in a quasi-equilibrium.

We denote the average value of the dependent variables over the selected interval as 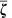, such that,


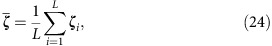


where **ζ**_*i*_ now denotes the true values of the variables in a particular year. Since, the annual values are assumed to be independent and identically distributed conditional on the linear model parameters, we then have,


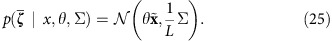


We assume that after this averaging step measurement error is negligible compared with the other sources of uncertainty.

For model comparison, we require the predicted distribution of the isotope measurements alone. This can be obtained by simple marginalization. We partition 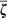 and the parameter matrices into isotope and sea ice retreat components,


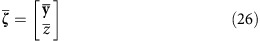



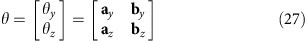



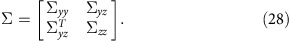


Using standard Gaussian density identities, the predicted distribution for the isotope measurements is then simply^47^,


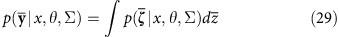



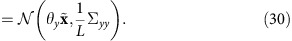


Using this basic formulation, models trained on the with-WAIS and without-WAIS simulation data both assign very small likelihoods to the measured isotope values. The problem is that neither model predicts the isotope measurements to within the expected accuracy, since both are imperfect representations of the real system. However, we can still assess which is better by incorporating this error into the analysis. To this end, the observed vector of isotopes 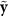 is modelled as the predicted value plus some error term, such that,


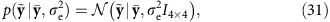


where *I*_4 × 4_ is the 4 × 4 identity matrix. Hence,


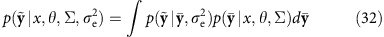



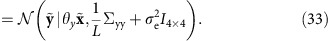


### Hypothesis testing

The standard mechanism for comparing two statistical models is to compute the marginal likelihood (also known as the model evidence) for each^47^. This is the probability assigned to the observed data by the model, averaging over all possible model parameter values,


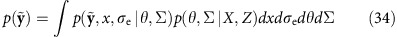



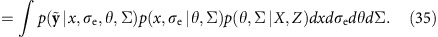


This cannot be evaluated analytically, so instead we approximate it numerically. The linear model parameter integrals are handled with Monte Carlo sampling. The remaining integrals over the heat flux and error scale variables may be handled using an empirical Bayes evidence approximation. Since the posterior distribution for these variables is sharply peaked, the prior probability density may be replaced with a point mass at the maximum likelihood value^47^,


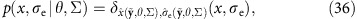


where


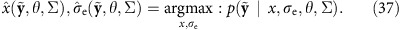


This also removes the necessity of specifying a prior distribution over *x* and *σ*_e_. Applying the two approximations, we obtain,


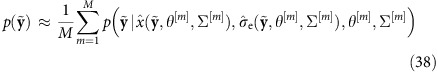



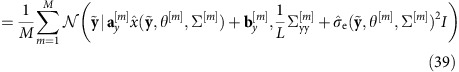


where 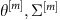 are sampled values of the linear model parameters drawn from the fitted posterior distribution. In our calculations, we used 1,000 Monte Carlo samples.

The average maximum likelihood values for heat flux are 72 and 51 W m^−2^, respectively, for the with-WAIS and without-WAIS models. Comparing the two scenarios, the likelihood ratio is 200 in favour of the WAIS being present (quoted to one significant figure), that is, the observed data is 200 times more likely using a model with the WAIS present than when the WAIS is removed. Moreover, the average error scale for the with-WAIS model is 0.6‰, compared with 1.9‰ for the without-WAIS model, indicating that larger error terms are needed in combination with the without-WAIS model to obtain the most likely system. These results strongly support the conclusion that the with-WAIS model is a more accurate representation of the ice core data. For the two scenarios, the probability of the with-WAIS model is 99.5%.

### Calculating the sea ice retreat

Taking into account the isotope measurements, knowledge about the corresponding average sea ice retreat is conveyed by the posterior distribution,


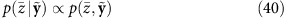



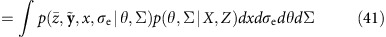



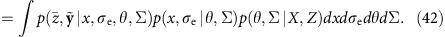


This is the probability distribution over the possible values for sea ice retreat conditional on the particular observed isotope measurements, but averaging over the possible values for the model parameters. As before, the integrals cannot be evaluated analytically, and numerical methods must be used.

Starting with the joint probability distribution over isotope and sea ice retreat, and applying the Monte Carlo and empirical Bayes approximations as before, we obtain,


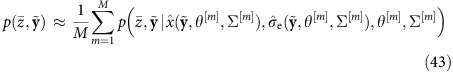



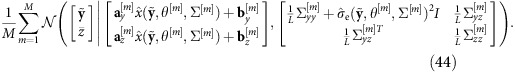


Finally, conditioning on the isotope measurements using standard Gaussian density identities^47^, the posterior distribution is approximated by,


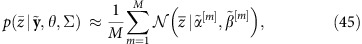


where,


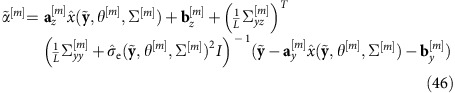



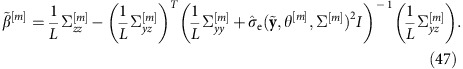


Our final approximation of the distribution is a Gaussian mixture, from which a mean value and credible intervals may be obtained. This provides us with the distribution of the average sea ice retreat over the *L* years in our window. There is an additional uncertainty of 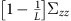 associated with each individual year due to the random annual variation.

For the with-WAIS scenario, we estimate the sea ice retreat during the LIG isotope maximum to be 65% (posterior mean). For the sea ice retreat in an arbitrary year, the 95% credible interval is [58, 72%]. For the average value of sea ice retreat over the 1,500 year period considered, the 95% credible interval is [61, 70%]. For the without-WAIS model, the same calculation similarly suggests an estimated best fit sea ice retreat to fit the observations during the LIG isotope maximum of 66% (posterior mean). However, the uncertainty band is more than three times larger than for the with-WAIS scenario. For the sea ice retreat in an arbitrary year, the 95% credible interval is [32, 87%]. For the average value of sea ice retreat over the 1,500 year period considered, the 95% credible interval is [32, 86%]. In the main text of the manuscript, we quote the posterior mean and the credibility interval for an arbitrary year during the 1,500 year period spanning the LIG isotope maximum for each scenario.

For the with-WAIS model, the maximum likelihood heat flux of 72 *Wm*^−2^ produces an annual averaged Southern Hemisphere sea ice area of ∼6 million km^2^ averaged over the whole 50 year simulation. This is equal to a globally averaged value of 0.82 W m^−2^, or ∼12% of the total radiative forcing in the representative concentration pathway 8.5 (RCP8.5) scenario between 2000 and 2100 (6.7 *Wm*^−2^). For context, Deser *et al.*^53^ calculate a value of 0.54 *Wm*^−2^, or 8% of the total radiative forcing in the RCP8.5 scenario, is required to change Arctic sea ice conditions from those simulated for the period 1980–1999 to those simulated for 2080–2099 under the RCP8.5 scenario using the CCSM4 model.

### Code availability

Access to the Met Office Unified Model source code is available under licence from the Met Office at http://www.metoffice.gov.uk/research/collaboration/um-collaboration. The code used to perform the statistical analysis is supplied as a supporting iPython notebook.

### Data availability

The climate model data is available on request from; http://www.bridge.bris.ac.uk/resources/simulations.

## Additional information

**How to cite this article:** Holloway, M.D. *et al.* Antarctic last interglacial isotope peak in response to sea ice retreat not ice-sheet collapse. *Nat. Commun.* 7:12293 doi: 10.1038/ncomms12293 (2016).

## Supplementary Material

Supplementary Information Supplementary Figures 1-6, Supplementary Tables 1-2, Supplementary Discussion and Supplementary References.

Supplementary Data 1 Analysis iPython notebook
