# Supplementary material for: Antarctic last interglacial isotope peak in response to sea ice retreat not ice-sheet collapse
Source: Nat Commun. 2016 Aug 16;7:12293. doi: 10.1038/ncomms12293 (PMC4990695; doi:10.1038/ncomms12293)
Supplement: Supplementary Information — Supplementary Figures 1-6, Supplementary Tables 1-2, Supplementary Discussion and Supplementary References. [file ncomms12293-s1.pdf]

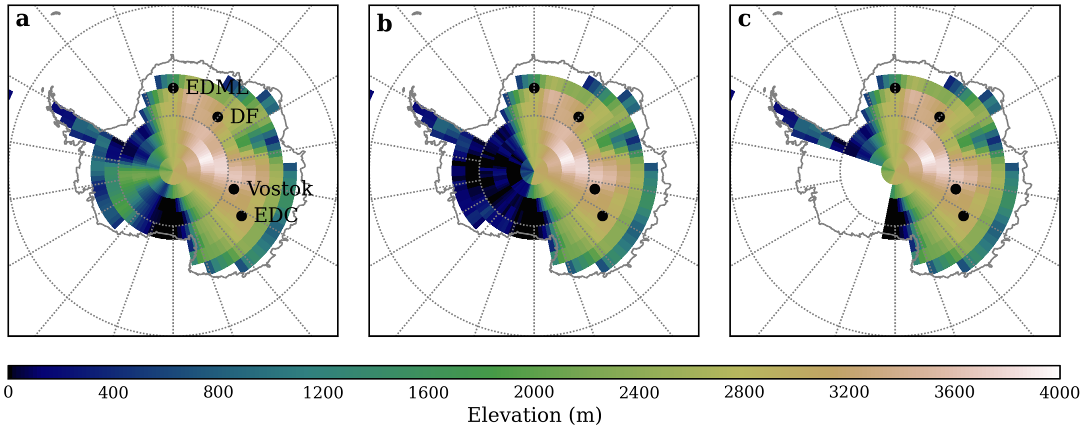

Supplementary Figure 1: Configurations of the West Antarctic Ice Sheet (WAIS) used for WAIS sensitivity experiments. (a) A modern WAIS configuration. (b) A remnant flat WAIS, at an elevation of 200 m. (c) The entire WAIS removed, and the exposed (submerged) bedrock replaced with a new region of ocean. The locations of the four ice core sites analysed in this study are marked with black circles and labelled in (a).

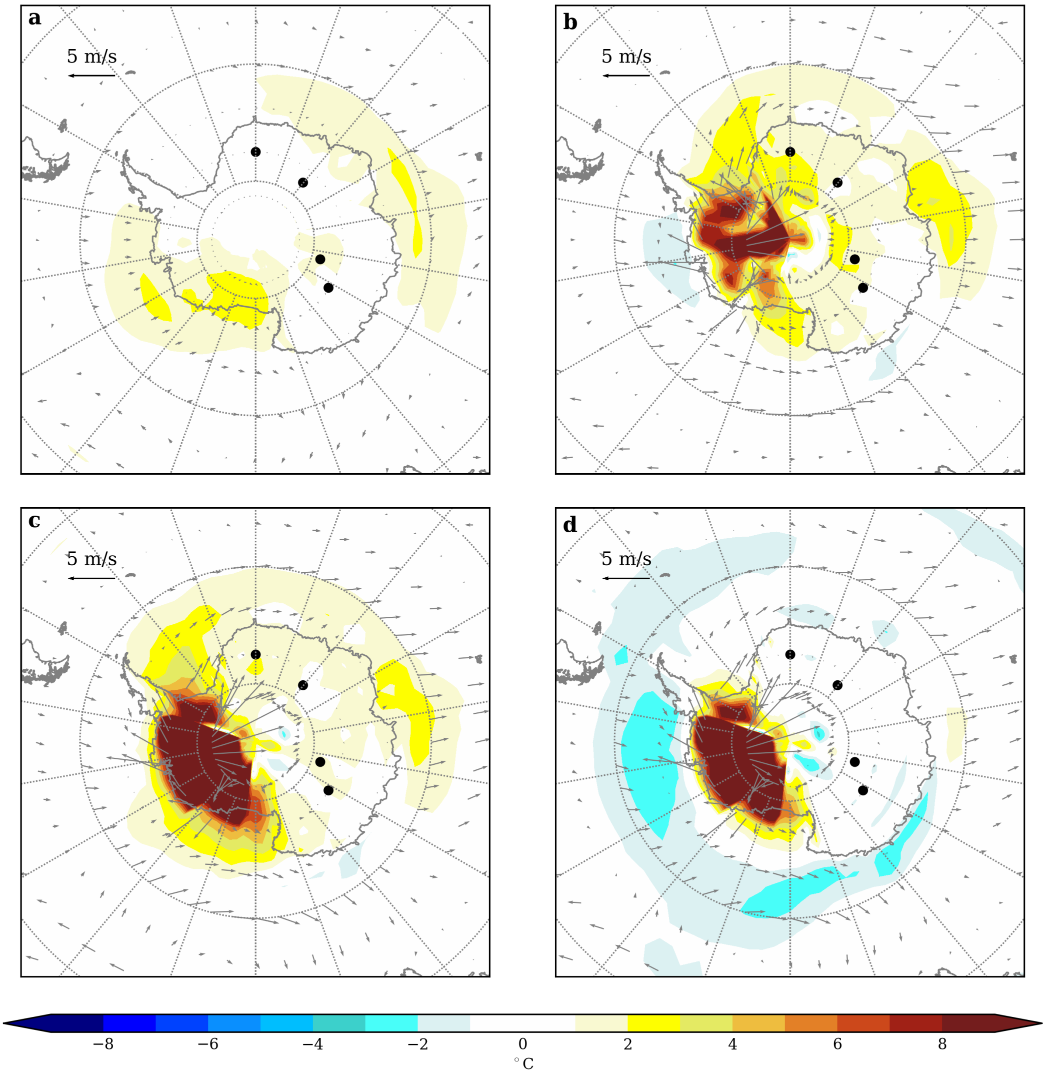

Supplementary Figure 2: Spatial pattern of surface air temperature and wind vector anomalies. Surface air temperature and wind vector anomalies (LIG- PI) for 128 ka simulations with (a) a modern WAIS configuration, (b) the WAIS flattened, (c) the WAIS removed and replaced with a new region of ocean, and (d) the WAIS removed and meltwater added to the Southern Ocean. The locations of the four ice core sites analysed in this study are marked with black circles.

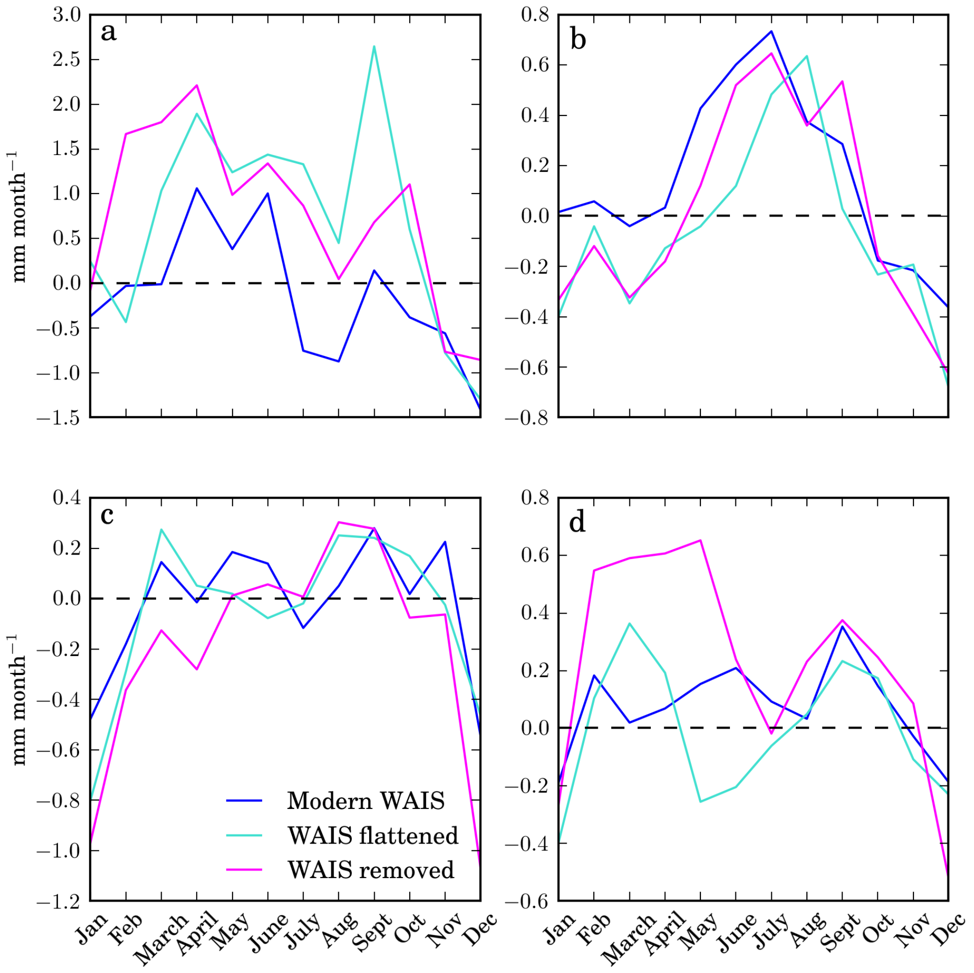

Supplementary Figure 3: Change in the seasonal cycle of precipitation (mm month^{-1}) at the ice core sites. (a) EPICA Dronning Maud Land (EDML), (b) Dome F, (c) Vostok, and (d) EPICA Dome C (EDC). Anomalies are calculated between 128 ka simulations using a modern WAIS (blue), a remnant flattened WAIS (turquoise) and with WAIS removed and replaced by ocean (magenta) compared to a pre-industrial simulation using a modern WAIS configuration.

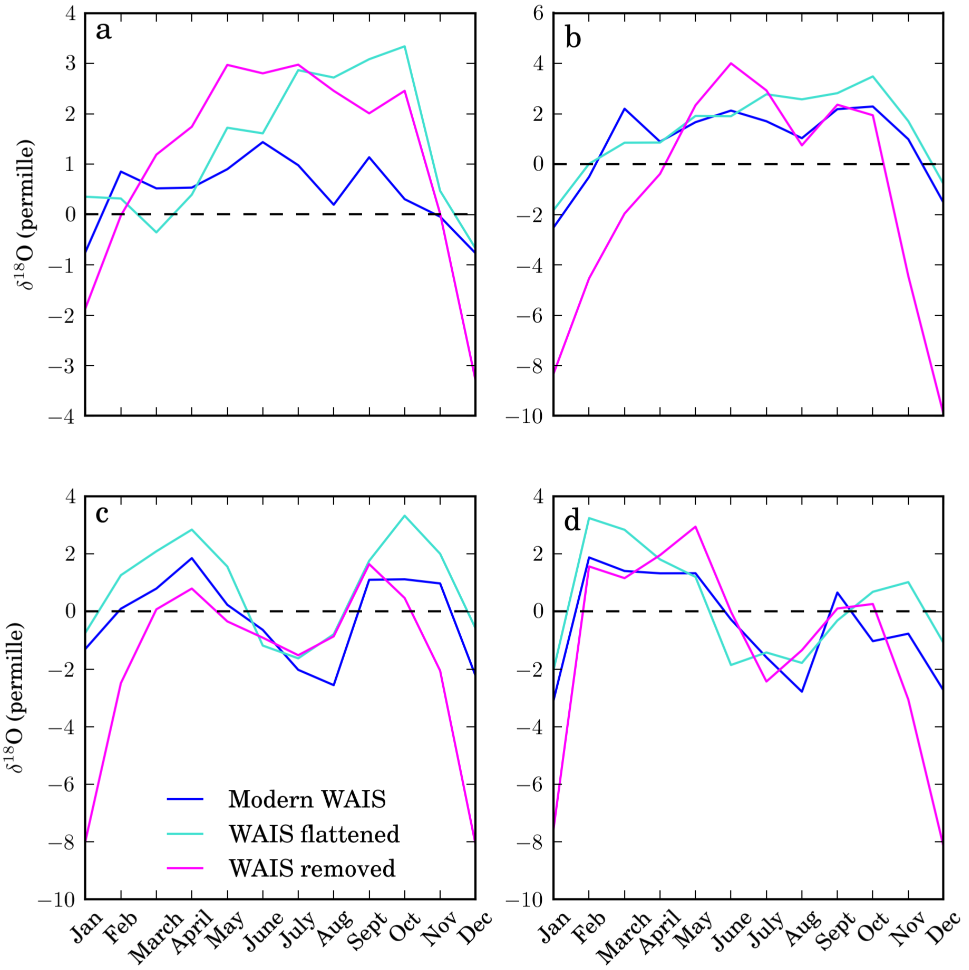

Supplementary Figure 4: Change in the seasonal cycle of $\delta^{18}O$ (‰) at the ice core sites. (a) EPICA Dronning Maud Land (EDML), (b) Dome F, (c) Vostok, and (d) EPICA Dome C (EDC). Anomalies are calculated between 128 ka simulations using a modern WAIS (blue), a remnant flattened WAIS (turquoise) and with WAIS removed and replaced by ocean (magenta) compared to a pre-industrial simulation using a modern WAIS configuration.

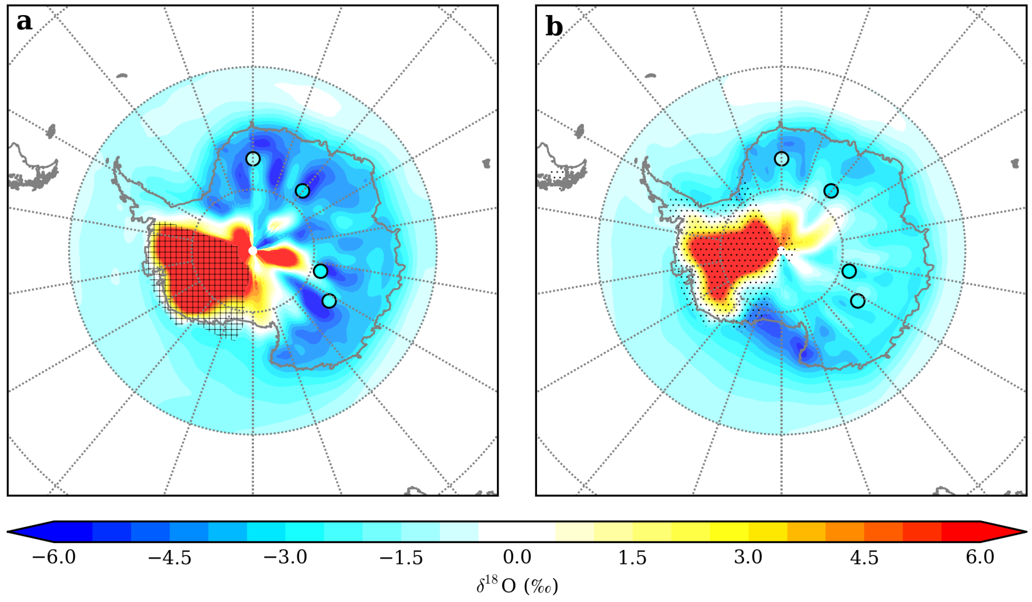

Supplementary Figure 5: Difference between sea ice retreat and WAIS collapse experiments. Difference in $\delta^{18}O$ (‰) between 128 ka experiments with a modern WAIS configuration and 65 % sea ice retreat and an experiment with (a) the WAIS removed and replaced with a new region of ocean (indicated by crosshatching) and (b) a remnant flat WAIS (indicated by stippling). Filled circles show the peak-to-trough $\delta^{18}O$ anomalies calculated for each ice core record between the LIG maximum and following minimum.

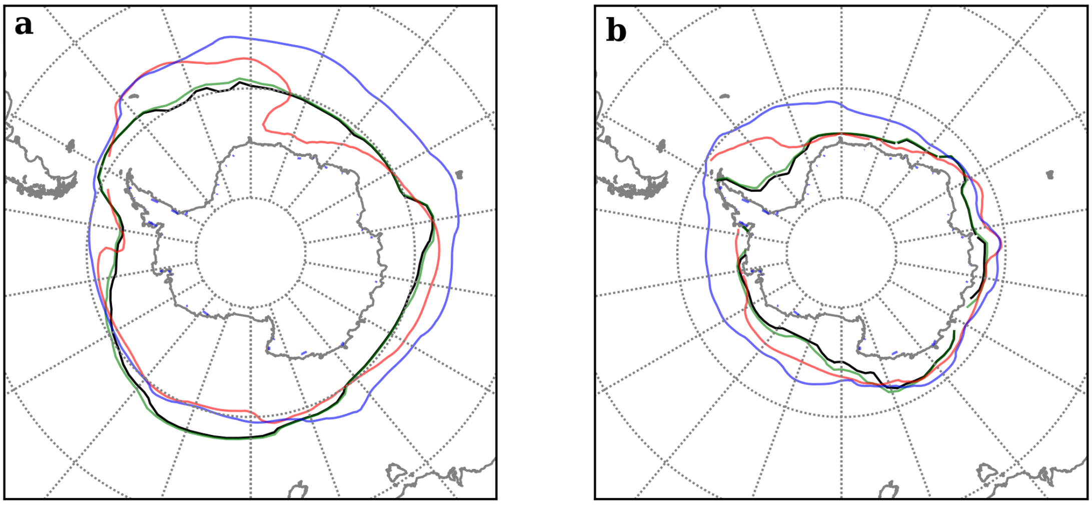

Supplementary Figure 6: Model comparison of last interglacial Antarctic sea ice extent. (a) September and (b) March sea ice extent simulated for the LIG from three coupled GCMs; HadCM3 125 ka simulation[4] (green), HadCM3 128 ka simulation (black), ECHAM5 125 ka simulation[5] (red) and CCSM3 125 ka simulation[6] (blue). Sea ice extent is defined by grid-cells with a sea ice fraction exceeding a threshold of 0.15.

Exp. ID	Orbit	CO_2	CH_4	N_2O	WAIS	FWF	SIF	ΔSI
	ka	ppmv	ppmv	ppmv		Sv (10^6 m^3 s^{-1})	W m^{-2}	%
PI	0	280	0.76	0.27	Modern	0	0	0
MOD-WAIS	128	275	0.707	0.266	Modern	0	0	-8
SUB-WAIS	128	275	0.707	0.266	Submerged	0	0	-9
FLAT-WAIS	128	275	0.707	0.266	Flat	0	0	-5
SUB-WAIS-SO	128	275	0.707	0.266	Submerged	0.4	0	15
MOD-WAIS-SI15	128	275	0.707	0.266	Modern	0	15	-32
MOD-WAIS-SI20	128	275	0.707	0.266	Modern	0	20	-37
MOD-WAIS-SI25	128	275	0.707	0.266	Modern	0	25	-43
MOD-WAIS-SI30	128	275	0.707	0.266	Modern	0	30	-48
MOD-WAIS-SI35	128	275	0.707	0.266	Modern	0	35	-51
MOD-WAIS-SI40	128	275	0.707	0.266	Modern	0	40	-54
MOD-WAIS-SI45	128	275	0.707	0.266	Modern	0	45	-52
MOD-WAIS-SI50	128	275	0.707	0.266	Modern	0	50	-55
MOD-WAIS-SI55	128	275	0.707	0.266	Modern	0	55	-57
MOD-WAIS-SI60	128	275	0.707	0.266	Modern	0	60	-61
MOD-WAIS-SI80	128	275	0.707	0.266	Modern	0	80	-66
MOD-WAIS-SI100	128	275	0.707	0.266	Modern	0	100	-71
MOD-WAIS-SI120	128	275	0.707	0.266	Modern	0	120	-76
SUB-WAIS-SI15	128	275	0.707	0.266	Submerged	0	15	-40
SUB-WAIS-SI20	128	275	0.707	0.266	Submerged	0	20	-45
SUB-WAIS-SI30	128	275	0.707	0.266	Submerged	0	30	-55
SUB-WAIS-SI35	128	275	0.707	0.266	Submerged	0	35	-58
SUB-WAIS-SI40	128	275	0.707	0.266	Submerged	0	40	-61
SUB-WAIS-SI45	128	275	0.707	0.266	Submerged	0	45	-64
SUB-WAIS-SI50	128	275	0.707	0.266	Submerged	0	50	-67
SUB-WAIS-SI55	128	275	0.707	0.266	Submerged	0	55	-68
SUB-WAIS-SI60	128	275	0.707	0.266	Submerged	0	60	-71
SUB-WAIS-SI80	128	275	0.707	0.266	Submerged	0	80	-76
SUB-WAIS-SI100	128	275	0.707	0.266	Submerged	0	100	-80
SUB-WAIS-SI120	128	275	0.707	0.266	Submerged	0	120	-84

Supplementary Table 1: List of experiments. Prescribed orbital parameters (ka), greenhouse gas concentrations (CO_2, CH_4, and N_2O; ppmv), West Antarctic Ice Sheet configuration (WAIS), meltwater flux (FWF; Sv), sea ice forcing (SIF; W m^{-2}) and the percentage change in September (winter) Antarctic relative to the pre-industrial (PI) simulation (ΔSI; %).

Model	Complexity	Period	Sept sia	March sia	Sept sia retreat
			10^6 km^2	10^6 km^2	%
Observations$_a$			20.14	5.13	
HadCM3	AOGCM$_b$	0 ka	22.46	4.48	
HadCM3	AOGCM	128 ka	20.73	4.88	7.72
HadCM3$_c$	AOGCM	125 ka	21.25	5.45	5.39
ECHAM5/MPI-OM$_d$	AOGCM	0 ka	23.22	6.83	
ECHAM5/MPI-OM$_d$	AOGCM	125 ka	20.4	6.94	12.14
CCSM3$_e$	AOGCM	125 ka	31.19	13.7	
CCSM3$_f$	AOGCM	PIG$_g$	24.67	7.48	
CCSM3$_f$	AOGCM	LIG$_h$	23.6	9.08	4.32
BERN3D$_f$	EMIC$_i$	PIG	12.13	8.15	
BERN3D$_f$	EMIC	LIG	11.52	8.1	5.01
CLIMBER$_f$	EMIC	PIG	21.42	2.99	
CLIMBER$_f$	EMIC	LIG	20.38	3.19	4.89
FAMOUS$_f$	EMIC	PIG	27.11	7.94	
FAMOUS$_f$	EMIC	LIG	26.28	8.59	3.04
LOVECLIM$_f$	EMIC	PIG	18.65	5.56	
LOVECLIM$_f$	EMIC	LIG	18.24	6.95	2.19
Multi-model mean			21.82	7.59	5.53

[a] Modern observational 5-day record from September 2014 (National Snow & Ice Data Center, 2014, https://nsidc.org).
[b] AOGCM: Atmosphere-Ocean General Circulation Model.
[c] Model data from Holloway et al. (2016)[4].
[d] Model data from Fischer et al. (2010)[5].
[e] Model data from Otto-Bliesner et al. (2013)[6].
[f] Model data from Bakker et al. (2014)[1].
[g] PIG; present interglacial (1.45-1.2 ka) (Bakker et al., 2014)[1].
[h] LIG; last interglacial (122.75-123 ka) (Bakker et al., 2014)[1].
[i] EMIC: Earth System Model of Intermediate Complexity.

Supplementary Table 2: Model comparison of last interglacial Antarctic sea ice area. Seasonal maximum (September) and minimum (March) sea ice area (sia; million km^2) for a range of climate models included in the most recent Paleoclimate Model Intercomparison Project Phase 3 for the Intergovernmental Panel for Climate Change 2013 report. Present day observations are shown, along with model results covering the present interglacial (PIG) and the last interglacial (LIG). Far right column shows the simulated September sea ice area retreat between the present and the last interglacial (%).

Supplementary Discussion

We compare the LIG sea ice response from a selection of models that participated in the LIG experiment as part of the Paleoclimate Model Intercomparison Project Phase 3 (PMIP3)[1,2] and the Past4Future project. As the modelled climate response across the early LIG is fairly consistent, we compile all available simulations covering this time period, even though some simulations differ from the original PMIP3 guidelines (also known as an 'ensemble of opportunity'[2]). The ensemble includes Atmosphere-Ocean GCMs that participated in the Coupled Model Intercomparison Project Phase 3 (CMIP3) and CMIP5 and a selection of Earth System Models of Intermediate Complexity (EMICs) that were evaluated in the IPCC AR5 report[3]. This ensemble is largely made up of time slice simulations centred on 125 ka (HadCM3[4], ECHAM5[5], and CCSM3[6]) and transient simulations covering 122.75-123 ka (CCSM3, BERN3D, CLIMBER, FAMOUS, LOVECLIM)[1] (Supplementary Table 2).

Comparing only the 125 ka and 122.75-123 ka simulations provides a multi-model mean September (winter) and March (summer) Antarctic sea ice extent of 21.88 and 7.89 x 10^6 km^2 respectively. HadCM3 simulates a September Antarctic sea ice area that sits within 0.5 % of the multi-model mean and the simulated March sea ice extent by HadCM3 is 1.88 x 10^6 km^2 below the multi-model mean. The HadCM3 pre-industrial estimates are within 16 and 5 % of the observed September and March sea ice extents respectively.

The Antarctic sea ice extents (using a concentration threshold of 15 %) for three models with LIG time slice simulations available; HadCM3, ECHAM5, and CCSM3, are shown in Supplementary Figure 6. HadCM3 and ECHAM5 agree well on the total area of September and March sea ice extents but show different spatial patterns, especially in the Atlantic sector. CCSM3 simulates a larger LIG sea ice extent in both winter and summer compared to HadCM3 and ECHAM5.

This comparison indicates that no model captures a substantial decrease in winter sea ice extent during the LIG relative to the present interglacial (PIG)[1]. The largest winter sea ice retreat between the LIG and the PIG is simulated by ECHAM5, reaching 12 % (Supplementary Table 2). The mean LIG-PIG winter sea ice retreat of all the models is 5.53 %. These results suggest that Southern Hemisphere sea ice may be too insensitive in PMIP3 models to warmer than present climates and that a sea ice retreat at 128 ka may provide a possible explanation for the observed ice core signal.

Bibliography

1. Bakker, P *et al*. Temperature trends during the Present and Last Interglacial periods - a multi-model-data comparison. *Quaternary Science Reviews* 99, 224-243, doi:10.1016/j.quascirev.2014.06.031 (2014).
2. Lunt, D. J. *et al*. A multi-model assessment of last interglacial temperatures. *Climate of the Past* 9, 699-717, doi:10.5194/cp-9-699-2013 (2013).
3. IPCC. *Climate Change 2013: The Physical Science Basis. Contribution of Working Group I to the Fifth Assessment Report of the Intergovernmental Panel on Climate Change*. Eds. Stocker, T.F. and Qin, D and Plattner, G and Tignor, M and Allen, S.K. and Boschung, J and Nauels, A and Xia, Y and Bex, V and Midgley, P.M. Cambridge University Press, Cambridge, United Kingdom and New York, NY, USA, doi:10.1017/CBO9781107415324, 1535 pp (2013).
4. Holloway, M. D., Sime, L. C., Singarayer, J. S., Tindall, J. C. & Valdes, P. J. Reconstructing Paleosalinity from $\delta^{18}O$: Coupled model simulations of the Last Glacial Maximum, Last Interglacial and Late Holocene. *Quaternary Science Reviews* 131, 350-364, doi:10.1016/j.quascirev.2015.07.007 (2016).
5. Fischer, N. & Jungclaus, J. H. Effects of orbital forcing on atmosphere and ocean heat trans- ports in Holocene and Eemian climate simulations with a comprehensive Earth system model. *Climate of the Past* 6, 155-168 (2010).
6. Otto-Bliesner, B. L. *et al*. How warm was the last interglacial? New model-data comparisons. *Philsophical Transactions of the Royal Society A* 371, 20130097, doi: 10.1098/rsta.2013.0097 (2013).
